# 

**Published:** 1891-06

**Authors:** 


### CONTENTS—JUNE, 1891.—VOL. VI., NO. 12.

ORIGINAL COMMUNICATIONS—
Clinical Notes from Practice. R. C. Hodges, M. D. . . . . . 493
Acute Hyperthrophy of the Mammary Glands. T. J. Crofford, M. D. . . . . . 497
Urethral Caruncle, their Treatment. J. W. Hamilton, M. D. . . . . . 503
Apocynum Cannabinum. W. T. Richmond, M. D. . . . . . 505
CORRESPONDENCE—
An International Medical Congress . . . . . 507
SOCIETY NOTES—
An Inter-Continental American Medical Congress . . . . . 509
Central Texas Medical Association, 20th Quarterly Session . . . . . 511
EDITORIAL—
The State Lunatic Asylum at Austin . . . . . 513
"Our Noble Profession" . . . . . 516
The Great Medical Temperance Movement . . . . . 518
The "Decline and Fall Off" . . . . . 520
Efficient Work . . . . . 521
SELECTIONS—
Report of Committee on Cabinet Appointment of Secretary of Health . . . . . 522
MEDICAL NEWS AND MISCELLANY . . . . . 527

